# Identifying early predictive and diagnostic biomarkers and exploring metabolic pathways for sepsis after trauma based on an untargeted metabolomics approach

**DOI:** 10.1038/s41598-025-92631-3

**Published:** 2025-04-08

**Authors:** Yi Gou, Bo-Hui Lv, Jun-Fei Zhang, Sheng-Ming Li, Xiao-Ping Hei, Jing-Jing Liu, Lei Li, Jian-Zhong Yang, Ke Feng

**Affiliations:** 1 Department of Emergency Medicine, General Hospital of Ningxia Medical University, Yinchuan, 750003 Ningxia China; 2 The First Affiliated Hospital of Xinjiang Medical University, Urumqi, 830011 China; 3 School of Nursing, Guizhou Medical University, Guiyang, 550025 China

**Keywords:** Sepsis, Trauma, Metabolomics, Biomarkers, Pathways, Lipids, Metabolomics

## Abstract

Systemic inflammatory response syndrome (SIRS) and organ dysfunction make it challenging to predict which major trauma patients are at risk of developing sepsis. Additionally, the unclear pathogenesis of sepsis after trauma contributes to its high morbidity and mortality. Identifying early predictive and diagnostic biomarkers, as well as exploring related metabolic pathways, is crucial for improving early prevention, diagnosis, and treatment. This study prospectively analyzed plasma samples from patients with severe trauma collected between March 2022 and November 2023. Trauma patients were divided into two groups based on whether they developed sepsis within two weeks: the TDDS group (trauma patients who did not develop sepsis) and the TDS group (trauma patients who did develop sepsis). Plasma samples from the TDS group were collected at the time of sepsis diagnosis (Sepsis group). Metabolite concentrations were measured using ultrahigh-performance liquid chromatography-tandem mass spectrometry (UHPLC-MS/MS) through untargeted metabolomics. From the differential metabolites between the TDS and TDDS groups, we identified five significant metabolites (all area under the curve (AUC) ≥ 0.94) as early predictive biomarkers for sepsis after trauma: (1) docosatrienoic acid, (2) 7-alpha-carboxy-17-alpha-carboxyethylandrostan lactone phenyl ester, (3) sphingomyelin (SM) 8:1;2O/26:1, (4) N1-[1-(3-isopropenylphenyl)-1-methylethyl]-3-oxobutanamide, and (5) SM 34:2;2O. Furthermore, five significant metabolites (all AUC ≥ 0.85) were identified as early diagnostic biomarkers from the comparison between the TDS and TDDS groups: (1) lysophosphatidylcholine (LPC) O-22:1, (2) LPC O-22:0, (3) uric acid, (4) LPC O-24:2, and (5) LPC 22:0-SN1. 26 metabolites shared between two comparisons (TDS vs. TDDS and sepsis vs. TDS) were identified. Of which, 19 metabolites belong to lipid metabolism. The top three metabolic pathways related to sepsis after trauma under the impact of severe trauma were: (1) glycerophospholipid metabolism, (2) porphyrin metabolism, and (3) sphingolipid metabolism. The top three metabolic pathways related to sepsis after trauma under the impact of infection were: (1) caffeine metabolism, (2) biosynthesis of unsaturated fatty acids, and (3) steroid hormone biosynthesis. Our study identified early predictive and diagnostic biomarkers and explored metabolic pathways related to sepsis after trauma. These findings provide a foundation for future research on the onset and development of sepsis, facilitating its early prevention, diagnosis, and treatment based on specific metabolites and metabolic pathways.

## Introduction

Trauma represents a prominent global public health concern and ranks as the fourth leading cause of mortality among the youth in China^1^. Sepsis, defined as life-threatening organ dysfunction arising from a dysregulated host response to infection, persists as the principal cause of fatality among trauma patients after the first week^2,3^. In severely traumatized patients, the premature release of damage-associated molecular patterns (DAMPs) triggers systemic inflammation, which, in turn, elicits post-traumatic immunosuppression, thereby augmenting the susceptibility to sepsis^4,5^. Additionally, disruption of mechanical barriers and invasive medical procedures further elevate the risk of infection in trauma patients^6^. Although sepsis-related mortality has declined over the past two decades, the incidence and fatality rates of post-traumatic sepsis remain high^7,8^. Post-traumatic sepsis prolongs hospital stays and elevates mortality, with the fatality rate ranging from 20–40%^6,9–12^. Early and accurate diagnosis, coupled with prompt and efficacious treatment, is essential for reducing the incidence and mortality of sepsis^13^. However, predicting which patients are at risk of developing sepsis following trauma remains a formidable challenge^14^. Trauma patients frequently present with SIRS, which poses a tough challenge in differentiating post-traumatic sterile inflammation from sepsis^15^. Additionally, the employment of blood cultures for the early detection of sepsis is constrained by result acquisition delays and the potential for false negatives, thereby diminishing their efficacy in promptly identifying sepsis^16^. Furthermore, severe trauma patients tend to suffer from organ dysfunction, complicating the determination of whether the dysfunction was caused by trauma or sepsis^17–19^. The obscure pathogenesis of post-traumatic sepsis constitutes another crucial factor contributing to its high morbidity and mortality. Consequently, identifying early predictive biomarkers to single out high-risk patients, along with diagnostic biomarkers for the timely detection of sepsis, and exploring the metabolic pathways associated with post-traumatic sepsis are imperative for enhancing the prevention, diagnosis, and treatment of this condition. Hong-Xiang Lu et al.^20^. developed and validated a traumatic sepsis score, with the areas under the receiver operating characteristic curve (AUC) being 0.799 and 0.790 for the training and validation datasets, respectively. Nevertheless, its predictive value remains to be confirmed in multi-center studies carried out in other hospitals or countries. Gian Paolo Castelli et al.^21^. explored the diagnostic value of procalcitonin (PCT) in septic complications subsequent to major trauma. They found that, compared with the concentrations measured one day prior to sepsis diagnosis, trauma patients demonstrated an early and substantial increase in PCT at the onset of sepsis (AUC = 0.787). Nevertheless, elevated PCT levels are prevalent among patients with multiple traumas, rendering it arduous to differentiate between infectious and non-infectious acute inflammation based on PCT levels^22^. Gene expression levels were also utilized to discriminate between infection and sterile inflammation and identify trauma patients at risk of sepsis^23^. Hongxiang Lu et al.^24^. identified that the rs2232618 polymorphism is associated with a high risk of sepsis. They further discovered that the enhancer-region polymorphism rs10865710 confers susceptibility to traumatic sepsis^25^. Although screening for susceptibility genes can contribute to guiding individualized therapy, its time-consuming nature and limited utility in early prediction may curtail its clinical application in post-traumatic sepsis.

Metabolomics, an emerging and rapidly evolving field in systems biology, enables the comprehensive quantification of all detectable small molecules, chemicals, and metabolites within a given sample^26^. Metabolites mirror endogenous processes, including genetic variation and transcriptional regulation, along with the impact of exogenous or environmental exposures^27^. Thus, metabolomics can be harnessed to explore the pathological mechanisms underlying diseases and to identify biomarkers with high sensitivity and specificity for evaluating disease risk and prognosis^28–30^. Swarnima Pandey et al.^31^. reported that gender serves as a crucial mortality biomarker for septic shock; females are associated with an anti-inflammatory response, while males are associated with a pro-inflammatory one. Their research may contribute to the identification of novel treatment targets in gender medicine. Regrettably, trauma patients were not included in their study. Haobo Bai et al.^32^. reported that serum bone morphogenetic protein 9 (BMP9) concentrations upon admission were associated with 28-day mortality. Septic patients with a higher risk of death had lower BMP9 levels. Further experiments demonstrated that BMP9 treatment enhanced the outcome in mice with sepsis. Their findings imply that BMP9 can serve as a biomarker for classifying patients, bearing independent prognostic significance. Despite these advancements, the incidence and mortality of sepsis have not been significantly diminished^31^. Therefore, it remains imperative to screen for early predictive and diagnostic biomarkers of sepsis and to explore the pathogenesis of sepsis, particularly in post-traumatic sepsis.

There are two main platforms of metabolomics analysis, including nuclear magnetic resonance (NMR) and mass spectrometry (MS) spectroscopy. The advantages of NMR lie in its being a non-destructive and highly reproducible technique. It is capable of furnishing a high level of structural information within a short time while requiring minimal sample preparation^31,33^. Nevertheless, NMR has relatively lower sensitivity. This implies that potentially important compounds present at lower concentrations can be masked by larger peaks and, consequently, go unrecognized^34^. MS can reliably identify metabolites. In particular, when coupled with chromatographic separation methods, MS’s resolution ability will be enhanced. MS-based metabolomics has been growing in popularity on account of its high sensitivity, versatility, and the ability to obviate the requirement for chemical derivatization by means of new ionization techniques. The principal advantage of untargeted metabolomics resides in its capacity to uncover novel metabolites since it enables the simultaneous measurement of numerous metabolites within each sample^35^. Benjamin J. Blaise et al.^36^. used NMR to identify an early metabolic phenotype in trauma patients at ICU admission that was associated with subsequent sepsis development (AUC = 0.778). In a previous study, three potential biomarkers, succinic semi-aldehyde (AUC = 0.7744), uracil (AUC = 0.7367), and uridine (AUC = 0.8156), were identified through untargeted plasma metabolomics based on MS and may be applied to the clinical identification of high-risk trauma patients who are likely to develop sepsis^37^. However, since blood samples were not collected on the first day of sepsis onset, this study did not identify diagnostic biomarkers for sepsis following trauma. Prior studies regarding the early diagnostic biomarkers of post-traumatic sepsis, which were founded on metabolomics, typically collected blood samples from trauma patients on the first day following injury. Subsequently, the patients were classified into a trauma group and a sepsis group depending on whether they later developed sepsis. Eventually, diagnostic biomarkers were screened out by comparing the two groups. In fact, the diagnostic biomarkers acquired through this method are predictive biomarkers. The aim of this study was to find predictive metabolites that distinguish trauma patients who developed sepsis from those who did not develop sepsis, as well as diagnostic metabolites that identify sepsis after trauma, and to identify related pathways.

## Methods

### Study design and patient selection

This study was approved by the Ethics Committee of Ningxia Medical University General Hospital (KYLL-2021-938) and conducted in accordance with the ethical guidelines of the 1964 Declaration of Helsinki and its later amendments. Plasma samples were prospectively collected from patients with severe trauma between March 2022 and November 2023, as well as from non-trauma controls. Informed consent was obtained from all patients or their legal representatives in the case of incapacitated individuals.

Inclusion criteria for trauma patients were as follows: (1) Injury Severity Score (ISS) ≥ 16 or Abbreviated Injury Scale (AIS) ≥ 3 points; (2) Age ≥ 18 years; (3) Patients admitted to the emergency department within 24 h post-trauma; (4) A minimum hospital survival of 7 days. Exclusion criteria included, (1) pregnancy or lactation; (2) immunodeficient patients or those receiving immunosuppressive drugs, as well as tumor patients; (3) prior anti-infection treatment or blood transfusion before enrollment. Sepsis was defined according to the Third International Consensus Definitions for Sepsis and Septic Shock^2^. That is, sepsis is diagnosed based on infection + SOFA score of ≥ 2 points. The diagnostic criterion for infection is a positive result from etiological culture.Plasma samples of trauma patients who visited our hospital within 24 h after injury were obtained within the first hour of patient admission, and they were followed for up to 2 weeks. Based on the presence or absence of sepsis, trauma patients were divided into two groups: the TDDS group (trauma patients who did not subsequently develop sepsis, *n* = 50) and the TDS group (trauma patients who subsequently developed sepsis, *n* = 50). When it was determined that the patients in the TDS group had developed sepsis, their plasma samples were collected to form the sepsis group (*n* = 50) within 24 h of sepsis diagnosis. Based on the comparisons between the TDS group and the TDDS group, differential metabolites serve as predictive biomarkers. Based on the comparisons between the TDS group and the sepsis group, differential metabolites were screened to serve as diagnostic biomarkers. Metabolic pathways related to sepsis were explored based on the comparisons between (TDS group vs. TDDS group and Sepsis group vs. TDS group).

### Data collection

Comprehensive clinical data were obtained on the first day of emergency department admission and sepsis diagnosis, which included: (1) Trauma-related clinical data: Mechanism of injury, site of injury, presence of intestinal injury, open injuries, hemorrhagic shock, circulatory and respiratory dysfunction, liver and renal dysfunction, coagulation dysfunction, and whether patients underwent tracheal intubation or deep vein catheterization; (2) Trauma-related scores: Injury Severity Score (ISS), Abbreviated Injury Scale (AIS), Glasgow Coma Scale (GCS), and Sequential Organ Failure Assessment (SOFA) score; (3) Blood test data: White blood cell count (WBC), absolute neutrophil count (ANC), lymphocyte count (ALC), monocyte count (AMC), total bilirubin (TBIL), albumin (ALB), cholinesterase (CHE), blood urea nitrogen (BUN), creatinine (CREA), fibrinogen (FIB), D-dimer, oxygenation index (OI), lactic acid (LAC), and buffer excess (BE); (4) Demographics: Gender, age, and underlying diseases; (5) Other information: Temperature, time of sepsis onset, admission to ICU, and length of hospital stay (LOS).

## Metabolomics approach

### UHPLC-MS/MS analysis

Untargeted metabolomics analysis was performed using ultrahigh-performance liquid chromatography-tandem mass spectrometry (UHPLC-MS/MS) on a Vanquish UHPLC system (Thermo Fisher, Germany) coupled with an Orbitrap Q Exactive TM HF mass spectrometer or Orbitrap Q Exactive TM HF-X mass spectrometer (Thermo Fisher, Germany). The samples were injected onto a Hypersil Gold column (100 × 2.1 mm, 1.9 μm) using a 12-minute linear gradient at a flow rate of 0.2 mL/min. The eluents used for both positive and negative polarity modes were eluent A (0.1% formic acid in water) and eluent B (methanol). The gradient was set as follows: 2% B for 1.5 min, 2–85% B for 3 min, 85–100% B for 10 min, 100–2% B for 10.1 min, and 2% B for 12 min. The mass spectrometer was operated in both positive and negative polarity modes with a spray voltage of 3.5 kV, capillary temperature of 320 °C, sheath gas flow rate of 35 psi, auxiliary gas flow rate of 10 L/min, S-lens RF level of 60, and auxiliary gas heater temperature of 350 °C.

### Data processing and metabolite identification

The raw data from UHPLC-MS/MS analysis was processed using Compound Discoverer 3.3 (CD3.3, Thermo Fisher) to perform peak alignment, peak picking, and quantification of each metabolite. Normalized data were used to predict molecular formulas based on additive ions, molecular ion peaks, and fragment ions. These peaks were matched with the mzCloud (https://www.mzcloud.org/), mzVault, and MassList databases to obtain accurate qualitative and relative quantitative results. The quantification results were normalized as follows: sample raw quantitation value/(sum of sample metabolite quantitation values/the sum of QC1 sample metabolite quantitation values) to obtain relative peak areas. Metabolites with coefficient of variation (CV) values greater than 30% in QC samples were excluded. The final metabolite identification and relative quantification results were obtained after this filtering.

### Statistical analysis

Statistical analyses were performed using IBM SPSS (version 26.0). Continuous variables were expressed as mean ± standard deviation for normally distributed data and as median (interquartile range, 25–75%) for non-normally distributed data. Independent sample t-tests were used for normally distributed data, while the Mann-Whitney test was used for non-normally distributed data. Categorical variables were presented as numbers and percentages, and Pearson’s χ2 test or Fisher’s exact test was used for group comparisons. Analysis of covariance (ANCOVA) was used to adjust for variables such as ISS, GCS, SOFA, WBC, temperature, and oxygenation index. For all analyses, a p-value < 0.05 was considered statistically significant.

Partial least squares discriminant analysis (PLS-DA), a commonly used classification method that combines regression models with dimensionality reduction and discriminant analysis of regression results, was conducted using MetaboAnalyst 6.0 (https://www.metaboanalyst.ca/) for a PLS-DA score plot to visualize the classification effect of the model and for calculating the variable importance in projection (VIP) score. The greater the separation of the two groups in the plot, the more significant the classification effect. In PLS-DA cross validation, R² (coefficient of determination) and Q² (predictive ability index) assess model performance. R² reflects the explanatory power of the model towards the data, with its value ranging from 0 to 1. A value closer to 1 indicates that the model can account for a larger proportion of the variance in the original data. Q² evaluates the predictive ability of the model through cross-validation, also spanning from 0 to 1. A high value indicates that the model fits the existing data well and has a strong predictive accuracy. Univariate analysis (t-test) was performed to determine statistical significance. Differential metabolites were identified based on VIP score > 1 and p-value < 0.05. Analysis of covariance for each significant metabolite was performed to adjust ISS, GCS, and SOFA. Heatmaps were generated using MetaboAnalyst 6.0 to show the intuitive visualization of discriminant metabolites between (TDS group vs. TDDS group and Sepsis group vs. TDS group). Pathway analysis was conducted using MetaboAnalyst 6.0 to identify biological pathways significantly associated with sepsis after trauma. Area under the curve (AUC) graphics were created using GraphPad Prism (version 8.3.0) to calculate the predictive and diagnostic potential of biomarkers.

## Results

### Patients’ characteristics

100 traumatic patients were included, of which 50 were traumatic patients who subsequently developed sepsis (TDS) and 50 were traumatic patients who did not subsequently develop sepsis (TDDS). The time of sepsis development was 1 –14 day [5 (4, 6) day], 46% of patients developed sepsis within 5 days after injury. Comparisons of the baseline and clinical characteristics between the TDDS and TDS groups are shown in Table 1. ISS, GCS, and SOFA were significantly higher in TDS group. There was no statistically significant difference in age, sex, co‑morbidities, and injury mechanism between the two groups. Comparisons of the baseline and clinical characteristics between the TDS and sepsis groups are shown in Table 2.

**Table 1:** A comparison of the baseline and clinical characteristics of TDDS group and TDS group.

Characteristics	TDDS group (*N* = 50)	TDS group (*N* = 50)	χ2/t/Z	*P*-value
Age	48.7 ± 16.6	51.0 ± 15.9	−0.707	0.481
Sex (male, %)	37(74.0)	42(84.0)	1.507	0.220
ISS	19.0(16.8–24.0)	29.0(20.0-38.8)	−4.889	<0.001
GCS	15.0(8.0–15.0)	7.00(5.0–15.0)	4.346	<0.001
SOFA	3.0(2.0–5.0)	5.5(4.0–7.0)	−4.955	<0.001
Heart rate (times/min)	96.7 ± 18.4	109.3 ± 23.2	−3.028	0.003
Systolic pressure (mmHg)	131.1 ± 27.2	123.3 ± 31.2	1.325	0.188
Diastolic pressure (mmHg)	79.5 ± 14.6	75.5 ± 19.9	1.153	0.252
Mean arterial pressure (mmHg)	96.7 ± 17.9	91.4 ± 22.9	1.282	0.203
Respiratory rate (times/min)	20.0(19.8–22.0)	21.0(20.0–25.0)	−1.185	0.239
Pulse oxygen saturation (%)	93.5(87.0–96.0)	94.0(85.8–98.0)	0.749	0.456
WBC (10^3^/mm^3^)	17.1(12.6–20.3)	17.4(12.3–24.5)	−0.651	0.517
ANC (10^3^/mm^3^)	14.6(11.4–17.0)	14.7(9.7–21.3)	−0.579	0.564
RBC (10^4^/mm^3^)	4.1(3.8–4.5)	3.8(2.9–4.3)	2.096	0.005
HGB (g/L)	125.5 ± 24.1	111.9 ± 27.6	2.615	0.010
CREA (umol/L)	67.1(56.1–79.9)	69.2(60.7–85.4)	−2.191	0.032
TBIL (mmol/L)	16.3 ± 8.2	12.7 ± 6.8	2.369	0.020
Alb (g/ml)	34.7(31.1–39.2)	32.9(23.8–38.0)	2.101	0.038
CHE (U/L)	6038.0(5185.5-6794.5)	5541.5(4126.3–6630.0)	2.202	0.030
GLU (mmol/L)	8.8 ± 3.1	10.8 ± 5.0	−2.453	0.016
FIB (g/L)	2.4(1.8–2.8)	1.7(1.4–2.8)	1.847	0.355
D-dimer (ug/mL)	35.6(9.7–43.2)	38.3(17.5–80.5)	−1.709	0.092
LAC (mmol/L)	2.7(1.8–3.5)	3.3(1.8–5.7)	−1.724	0.088
BE (mmol/L)	-4.1(-6.0-2.1)	-5.5(-8.6-3.0)	2.014	0.047
Invasive MV (n, %)	11(22.0)	44(88.0)	44.000	<0.001
Catheterization in DV (n, %)	15(30.0)	29(58.0)	8.536	0.003
Admission to ICU (n, %)	25(50.0)	47(94.0)	24.008	<0.001
Co‑morbidities (n, %)	16(32.0)	18(36.0)	0.123	0.726
Hemorrhagic shock (n, %)	12(30.0)	27(54.0)	8.167	0.004
Open wound (n, %)	29(58.0)	34(68.0)	1.073	0.300
Injury mechanism (n, %)				
Traffic accident	37(74.0)	36(72.0)	3.680	0.159
Fall	11(22.0)	7(14.0)		
Others	2(4.0)	7(14.0)		
Head and neck (n, %)	32(64.0)	40(80.0)	3.175	0.075
Face (n, %)	16(32.0)	14(28.0)	0.190	0.663
Breast (n, %)	42(84.0)	39(78.0)	0.585	0.444
Abdomen (n, %)	25(50.0)	22(44.0)	0.361	0.548
Respiration dysfunction (n, %)	42(84.0)	43(86.0)	0.078	0.779
Circulation dysfunction (n, %)	4(8.0)	15(30.0)	7.862	0.005
Liver dysfunction (n, %)	1(2.0)	6(12.0)	3.840	0.117
kidney dysfunction (n, %)	3(6.0)	15(30.0)	9.756	0.002
Coagulation dysfunction (n, %)	20(40.0)	37(74.0)	11.791	0.001
Nerve dysfunction (n, %)	9(18.0)	6(12.0)	0.706	0.401
LOS in ICU (d)	1.50(0.0-9.3)	15.5(9.8–27.0)	−7.219	<0.001
TLOS (d)	19.0(14.0–23,3)	28.0(22.8–38.5)	−4.115	<0.001

*ISS* injury severity score, *GCS* Glasgow coma scale, *SOFA* sequential organ failure assessment, *WBC* white blood cell, *ANC* absolute neutrophil count, *RBC* red blood cell, *HGB* haemoglobin, *CREA* creatinine, *TBIL* total bilirubin, *Alb* albumin, *CHE* cholinesterase, *GLU* glucose, *FIB* fibrinogen, *LAC* lactic acid, *BE* base excess, *MV* mechanical ventilation, *DV* deep vein, *LOS* length of stay, *TLOS* total length of stay.

**Table 2:** A comparison of the baseline and clinical characteristics of TDS group and sepsis group.

Characteristics	TDS group (*N* = 50)	Sepsis group (*N* = 50)	Z/t/χ2	*P*-value
WBC (10^3^/mm^3^)	17.4(12.3–24.5)	11.8(8.5–13.8)	−4.880	<0.001
ANC (10^3^/mm^3^)	15.3 ± 6.8	9.3 ± 3.7	6.074	<0.001
ALC (10^3^/mm^3^)	1.2(0.7–2.1)	0.8(0.6–1.2)	4.207	<0.001
AMC (10^3^/mm^3^)	1.1 ± 0.6	0.9 ± 0.4	3.546	0.001
RBC (10^4^/mm^3^)	3.8(2.9–4.3)	3.1(2.7–3.6)	3.118	0.003
HGB (g/L)	119.5(91.0-134.3)	95.5(85.5–111.0)	3.359	0.002
CREA (umol/L)	69.2(60.7–85.4)	69.5(56.1–80.2)	−2.129	0.033
GLU (mmol/L)	10.0(7.9–12.1)	7.4(6.6–8.6)	−3.755	<0.001
TBIL (mmol/L)	12.0(7.9–14.4)	28.0(19.8–42.6)	−6.063	<0.001
ALT (U/L)	40.4(29.9-100.7)	46.6(30.9-112.5)	−1.136	0.261
AST (U/L)	59.2 (42.7-122.4)	57.7(34.9–96.1)	−0.554	0.582
Alb (g/ml)	31.2 ± 9.3	29.2 ± 6.3	1.396	0.169
CHE (U/L)	5182.3 ± 1694.4	3930.8 ± 1081.3	−4.550	<0.001
FIB (g/L)	1.7(1.4–2.8)	4.8(3.9–5.7)	−11.201	<0.001
D-dimer (ug/mL)	38.3(17.5–80.5)	8.9(6.1–18.5)	4.058	<0.001
OI (mmHg)	281.0(185.3-353.3)	200.5(161.9-260.4)	2.945	0.005
Temperature (℃)	36.9 ± 0.6	38.4 ± 0.5	−14.702	<0.001
Circulation dysfunction (n, %)	15(30.0)	29(58.0)		0.532
Respiration dysfunction (n, %)	38(76.0)	48(96.0)		<0.001
Liver dysfunction (n, %)	6(12.0)	34(68.0)		0.308
kidney dysfunction (n, %)	6(12.0)	2(4.0)		<0.001
Coagulation dysfunction (n, %)	16(32.0)	26(52.0)		0.366

*WBC* white blood cell, *ANC* absolute neutrophil count, *ALC* absolute lymphocyte count, *AMC* absolute monocyte count, *RBC* red blood cell, *HGB* haemoglobin, *CREA* creatinine, *GLU* glucose, *TBIL* total bilirubin, *ALT* alanine transaminase, *AST* aspartate transaminase, *Alb* albumin, *CHE* cholinesterase, *FIB* fibrinogen, *OI* oxygenation index.

### Assessment of metabolic profiles

A total of 1573 metabolites were identified and quantified, with 1153 detected in the positive ionization mode and 420 in the negative ionization mode. Lipids, lipid-like molecules, organic acids derivatives, and organoheterocyclic compounds were the most prevalent metabolites.

### TDS vs. TDDS for early predictive biomarkers of sepsis after trauma

To screen for early predictive biomarkers of post-traumatic sepsis, we recruited trauma patients who presented to our hospital within 24 h post-injury. Venous blood samples were retrieved from the patients within one hour of admission. Subsequently, these patients were followed up for a two-week period. Depending on whether they developed post-traumatic sepsis, they were classified into the TDS group and the TDDS group. By comparing the two groups, differential metabolites were identified as early predictive biomarkers for post-traumatic sepsis, with the aim of promptly (within 24 h post-injury) identifying high-risk patients who are potentially prone to developing post-traumatic sepsis.

### Screening great potential metabolites to distinguish TDS group from TDDS group

**Fig. 1:**
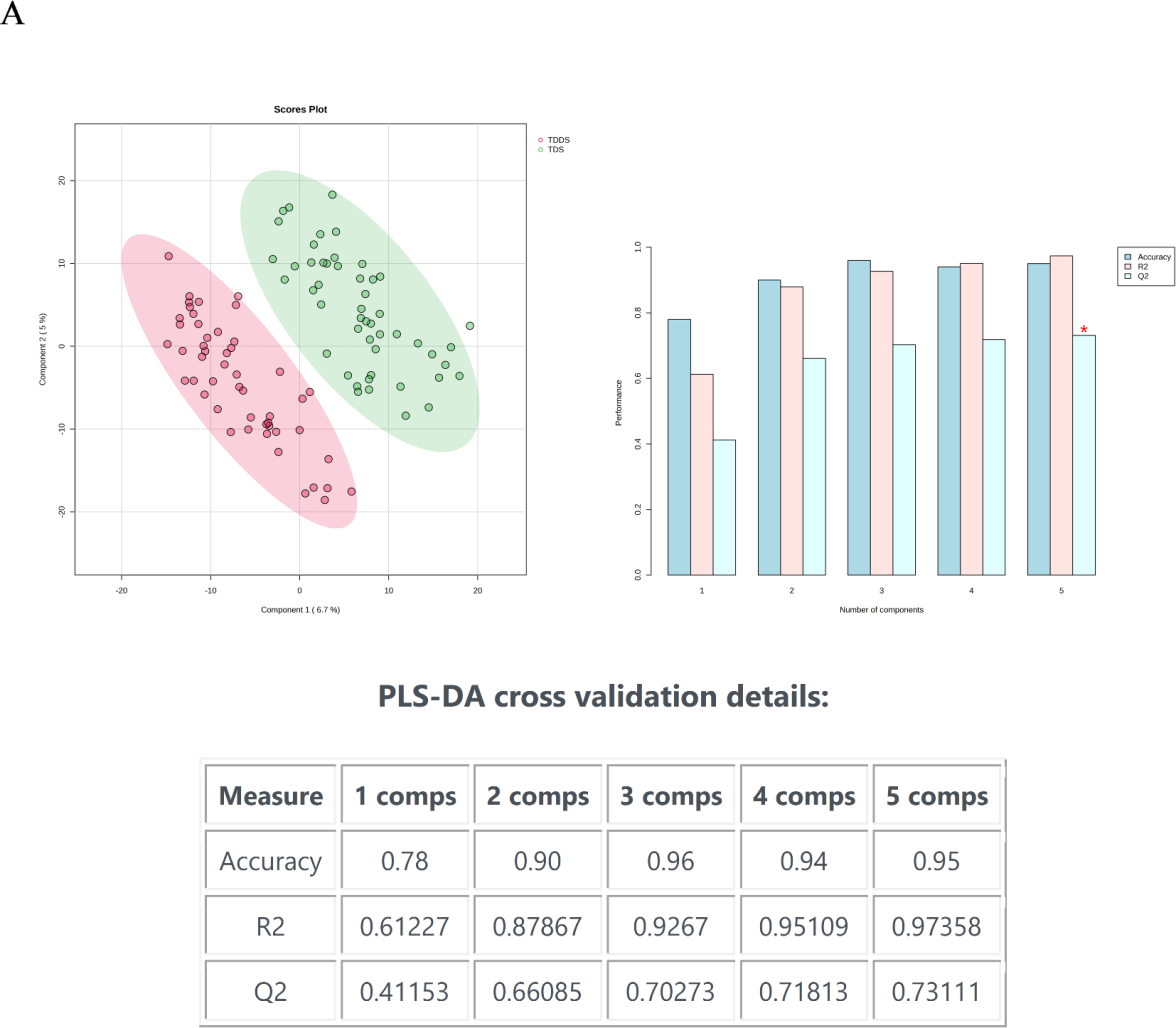

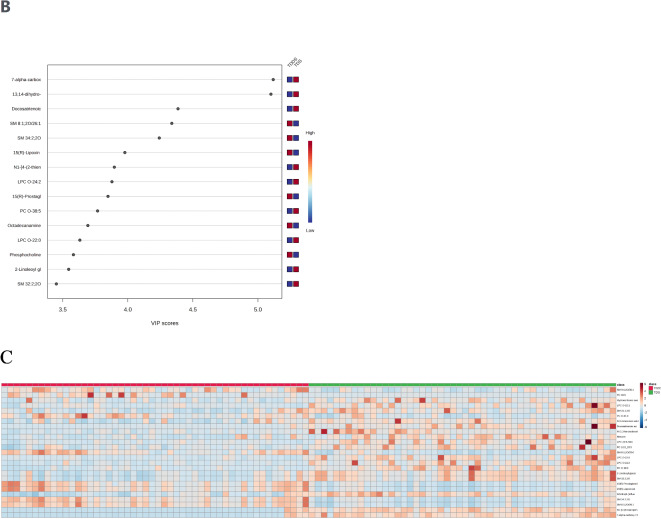
Statistical analysis of the data obtained from 50 traumatic patients in TDS group and 50 traumatic patients in TDDS group. (**A**) PLS‑DA 2D score plot for the discrimination of TDS group and TDDS group. (**B**) Important metabolites discriminating the two groups (Top 15). VIP score: the metabolites are responsible for discrimination TDS group and TDDS group. Metabolites with high VIP scores are more important in class separation. (**C**) Hierarchical heatmap for top‑25 discriminating metabolites between TDS and TDDS groups (red bar: TDDS group, green bar: TDS group).

We performed PLS-DA to distinguish between the TDS group and the TDDS group and calculate VIPscore. 2-dimensional score plotting showed that the two groups were distinguished by metabolic profiles (Fig. 1A). We chose 5 components which was achieved by cross validation method of PLSDA with R2 = 0.97, Q2 = 0.73, and accuracy of 0.95 (Fig. 1A). 409 metabolites with VIP scores>1 were screened out, the top 15 metabolites with high VIP scores are shown in Fig. 1B, illustrating that they have great potential to distinguish TDS group from TDDS group. A heatmap using Euclidean and T-test showed the intuitive visualization of discriminant metabolites between the TDS group and TDDS group (Fig. 1C).

### Screening early predictive biomarkers of sepsis after trauma

**Fig. 2:**
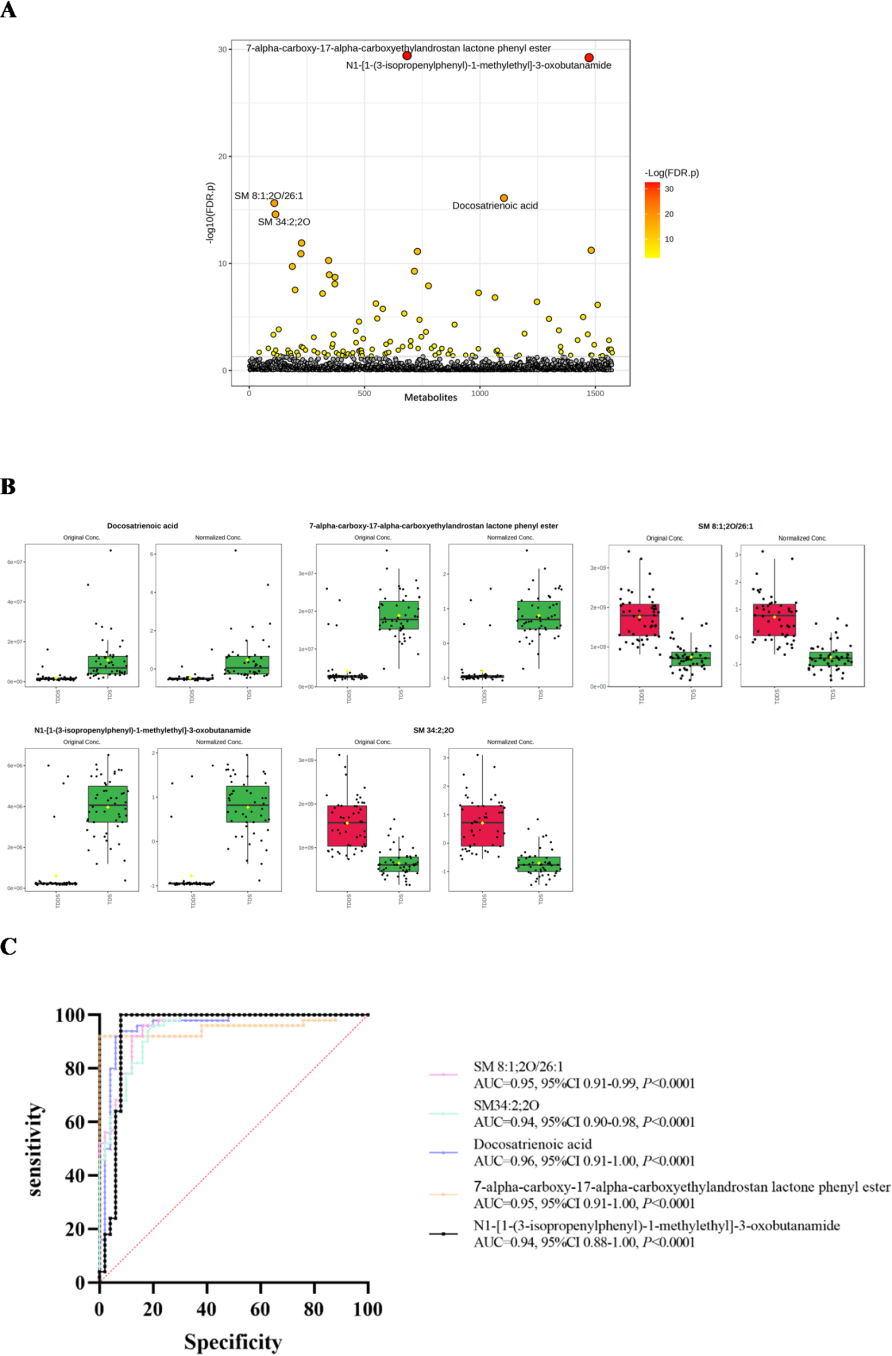
Screening early predictive biomarkers of sepsis after trauma. (**A**) T test between TDS group and TDDS group, circles represent metabolites, *Color gradient and circle size indicate the significance of the metabolites ranked by p‑value (grey: no significant, yellow: higher p‑value, red: lower p‑value). (**B**) The comparison of concentration between TDS group and TDDS group of five early predictive biomarker, (**C**) The areas under the ROC curves of five early predictive biomarker.

T test was performed between TDS group and TDDS group to screen metabolites with P-value<0.05,122 metabolites were significant, the top 5 metabolites were shown in Fig. 2A. 122 significantly differential metabolites were identified as early predictive biomarkers based on the criteria of VIP value > 1.0 and P value < 0.05. ROC curves were used to calculate the predictive potential of these metabolic biomarkers, The top 15 significantly different metabolites were shown in Table3. Finally, we selected 5 metabolites as early predictive biomarkers of sepsis after trauma, which were docosatrienoic acid, 7-alpha-carboxy-17-alpha-carboxyethylandrostan lactone phenyl ester, SM 8:1;2O/26:1, N1-[1-(3-isopropenylphenyl)-1-methylethyl]-3-oxobutanamide, and SM 34:2;2O among top 15 significantly different metabolites. Higher concentrations of docosatrienoic acid, 7-alpha-carboxy-17-alpha-carboxyethylandrostan lactone phenyl ester, and N1-[1-(3-isopropenylphenyl)-1-methylethyl]-3-oxobutanamide were observed in the TDS group as compared to the TDDS group (Fig. 2B). These 5 metabolites were still significant after adjusting for ISS, GCS, and SOFA. The areas under the ROC curves of the five early predictive biomarkers were shown in Fig. 2C.

**Table 3:** The top 15 significantly different metabolites to predict sepsis after trauma.

Metabolites	t.stat	*P* value	FDR	VIP	AUC
Docosatrienoic acid	−11.349	1.4754E-19	7.736E-17	4.3859	0.956
7-alpha-carboxy-17-alpha-carboxyethylandrostan lactone phenyl ester	−18.277	2.4517E-33	3.8565E-30	5.1176	0.952
SM 8:1;2O/26:1	11.069	5.8872E-19	2.3151E-16	4.3382	0.9504
N1-[1-(3-isopropenylphenyl)-1-methylethyl]-3-oxobutanamide	−18.004	7.6523E-33	6.0186E-30	5.1	0.942
SM 34:2;2O	10.539	8.2085E-18	2.5824E-15	4.2421	0.9412
15(R)-Lipoxin A4	−6.4268	6.5E-09	0.000000775	2.4381	0.912
LPC O-24:2	5.3749	0.000000621	0.000022	2.1446	0.9096
N,5-Bis(3-(trifluoromethyl)phenyl)oxazol-2-amine	−2.6831	0.0087126	0.037343	1.1863	0.8988
15(R)-Prostaglandin D2	9.2674	4.7113E-15	1.2351E-12	3.9775	0.8976
SM 32:2;2O	−7.29	8.0242E-11	8.4147E-09	3.4511	0.88
2-Linoleoyl glycerol	−7.6059	1.7413E-11	1.9564E-09	3.5459	0.8784
Phosphocholine	7.7318	9.4329E-12	1.1414E-09	3.5824	0.8764
PC O-38:5	−8.4082	3.3876E-13	5.3286E-11	3.7677	0.876
Octadecanamine	8.1283	1.3496E-12	1.9299E-10	3.6933	0.872
LPC O-22:0	−7.9052	4.0386E-12	5.2939E-10	3.6317	0.87

### Sepsis vs. TDS for early diagnostic biomarkers of sepsis after trauma

**Fig. 3:**
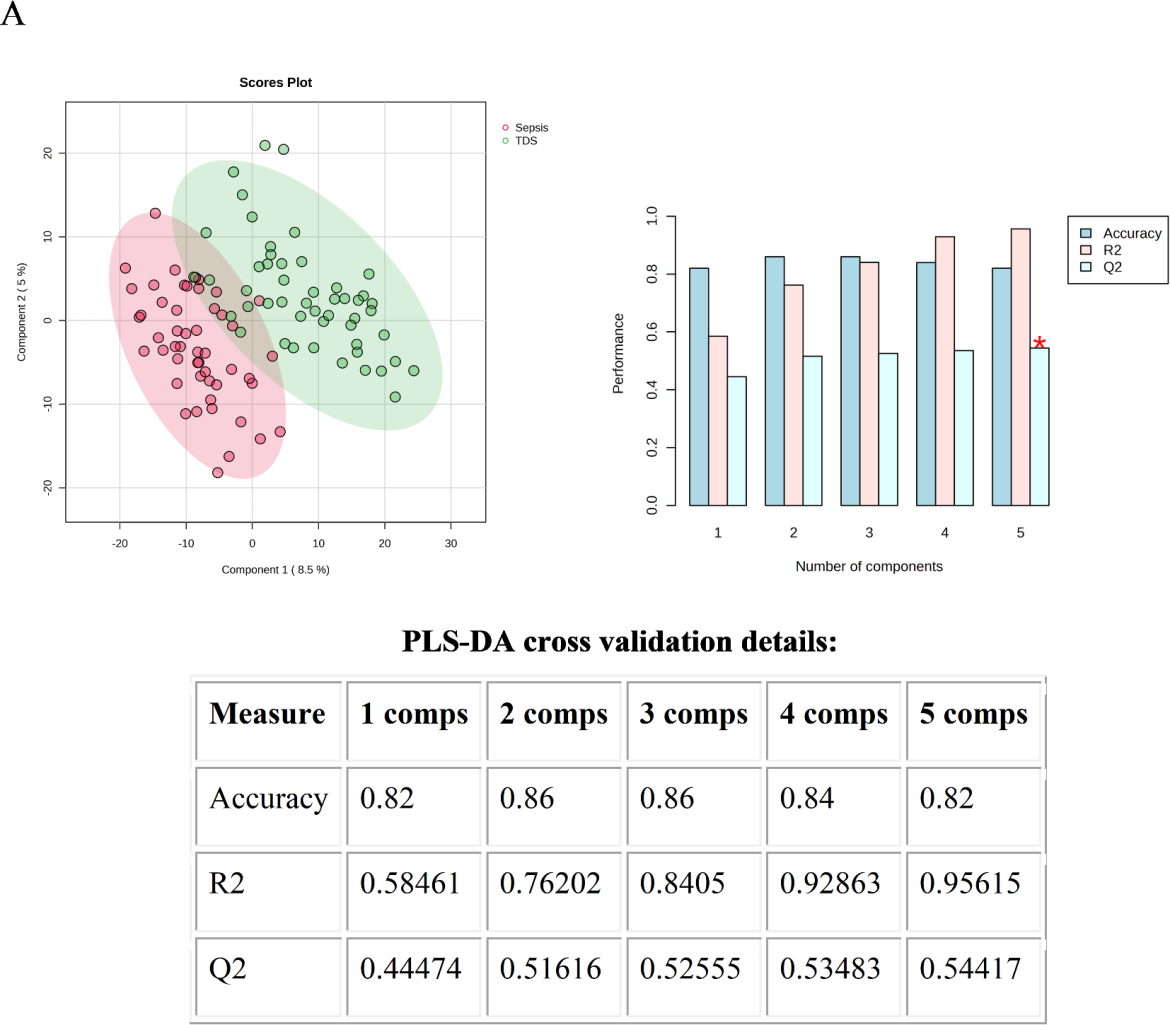

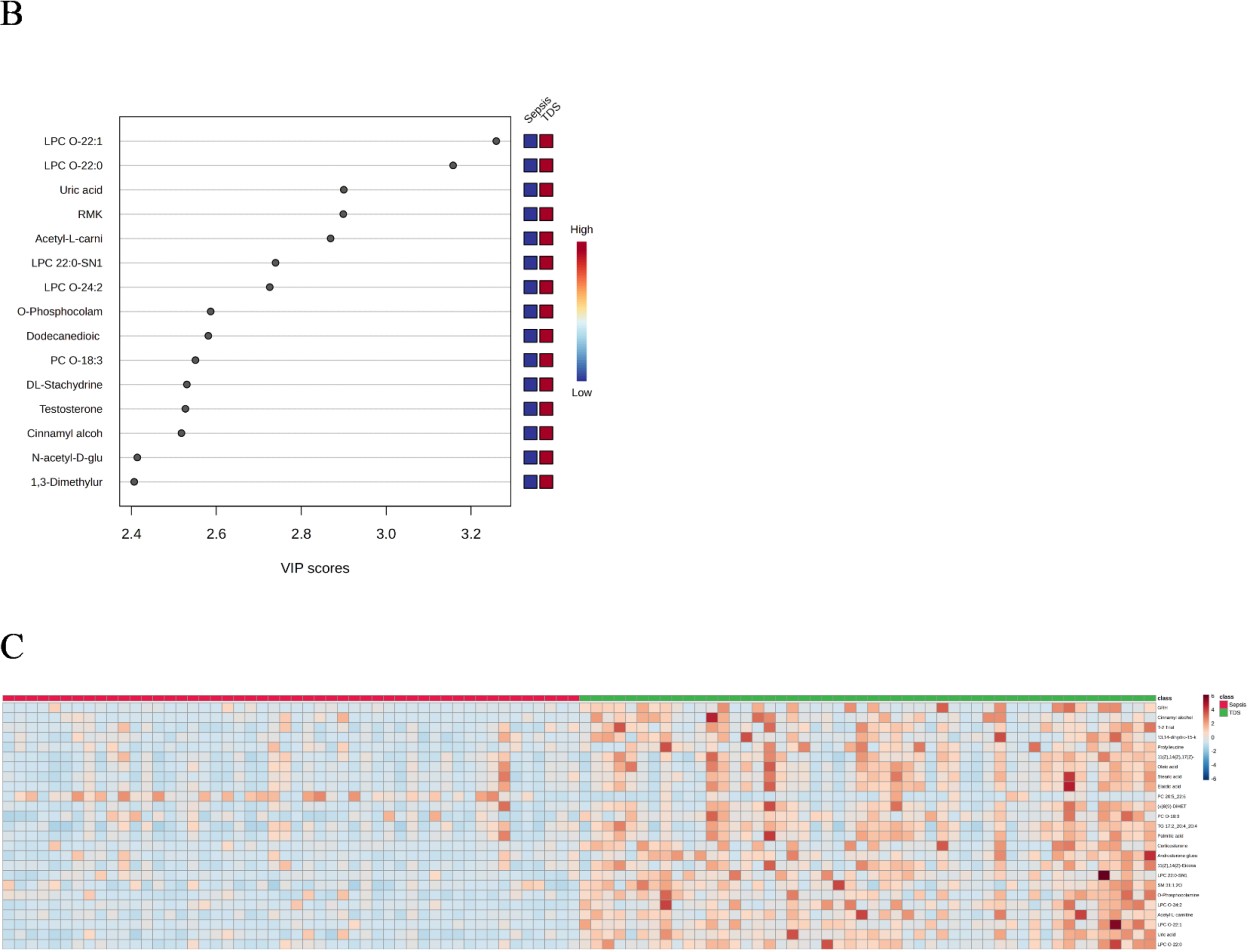
Statistical analysis of the data obtained from 50 traumatic patients in TDS group and 50 traumatic patients in Sepsis group. (**A**) PLS‑DA 2D score plot for the discrimination of Sepsis group and TDS group. (**B**) Important metabolites discriminating the two groups (Top 15). VIP score: the metabolites are responsible for discrimination Sepsis group and TDS group. Metabolites with high VIP scores are more important in class separation. (**C**) Hierarchical heatmap for top‑25 discriminating metabolites between Sepsis and TDS groups (red bar: Sepsis group, green bar: TDS group).

Venous blood was collected within 24 h after the patients in the TDS group were diagnosed with sepsis (sepsis group). By the comparison between before and after the occurrence of sepsis, early diagnostic biomarkers for post-traumatic sepsis were screened. PLS-DA was performed to distinguish between the Sepsis group and the TDS group and calculate VIP score. 2-dimensional score plotting showed that the two groups were distinguished by metabolic profiles (Fig. 3A). We chose 5 components which was achieved by cross validation method of PLSDA with R2 = 0.96, Q2 = 0.54, and accuracy of 0.82 (Fig. 3A). 510 metabolites with VIP scores>1 were screened out, the top 15 metabolites with high VIP scores are shown in Fig. 3B, illustrating that they have great potential to distinguish Sepsis group from TDS group. A heatmap using Euclidean and T-test showed the intuitive visualization of discriminant metabolites between the Sepsis group and TDS group (Fig. 3C).

### Early diagnostic biomarkers for sepsis after trauma

**Fig. 4:**
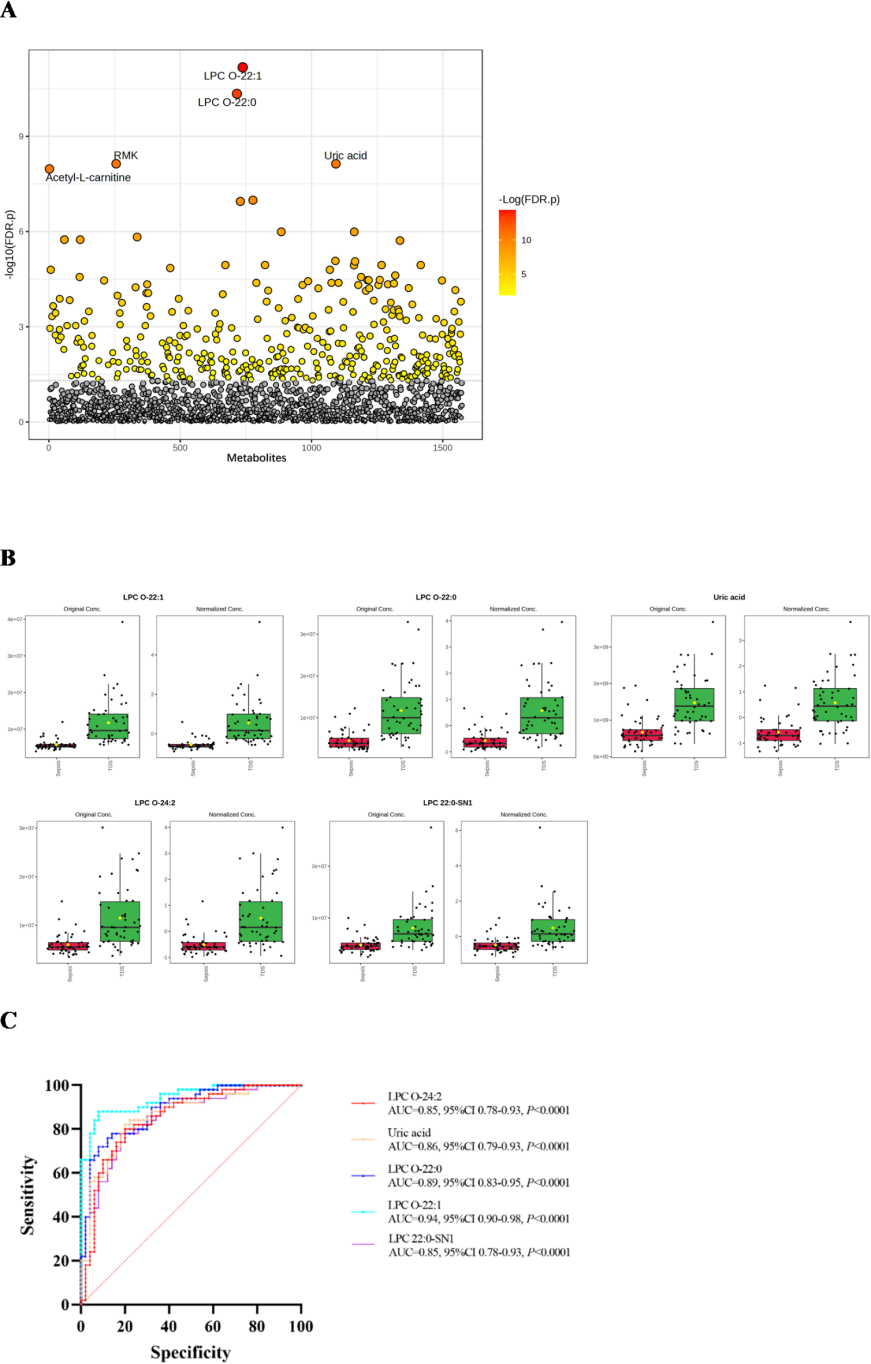
Screening early diagnostic biomarkers of sepsis after trauma. (**A**) T test between Sepsis group and TDS group, circles represent metabolites, *Color gradient and circle size indicate the significance of the metabolites ranked by p‑value (grey: no significant, yellow: higher p‑value, red: lower p‑value). (**B**) The comparison of the concentration of five early diagnostic biomarkers between Sepsis group and TDS group, (**C**) The areas under the ROC curves of five early diagnostic biomarkers.

T test was performed between Sepsis and TDS group to screen metabolites with P-value<0.05, 373 metabolites were significant, the top 5 metabolites were shown in Fig. 4A. 373 differential metabolites were identified as early diagnostic biomarkers based on the criteria of VIP value > 1.0 and P value < 0.05. ROC curves were used to calculate the diagnostic potential of these biomarkers, The top 15 significantly different metabolites were shown in Table 4. Finally, we selected top 5 differential metabolites as early diagnostic biomarkers of sepsis after trauma, which were LPC O-22:1, LPC O-22:0, Uric acid, LPC O-24:2, and LPC 22:0-SN1. Lower concentrations of LPC O-22:1, LPC O-22:0, Uric acid, LPC O-24:2, and LPC 22:0-SN1 were observed in Sepsis group as compared to the TDDS group (Fig. 4B). The areas under the ROC curves of the five early diagnostic biomarkers were shown in Fig. 4C.

**Table 4:** The top 15 significantly different metabolites to diagnose sepsis after trauma.

Metabolites	t.stat	*P* value	FDR	VIP	AUC
LPC O-22:1	−9.2872	4.2669E-15	6.7118E-12	3.2594	0.942
LPC O-22:0	−8.7639	5.7983E-14	4.5603E-11	3.1578	0.8896
Uric acid	−7.5969	1.8188E-11	7.3296E-09	2.9003	0.8624
LPC O-24:2	−6.9069	5.0004E-10	1.1237E-07	2.7259	0.852
LPC 22:0-SN1	−6.9583	3.9175E-10	1.027E-07	2.7395	0.8512
TG 8:0_8:0_18:3	−0.9282	0.0042393	0.023154	1.3513	0.8496
Acetyl-L-carnitine	−7.4689	3.3837E-11	1.0645E-08	2.8692	0.846
Testosterone	−6.1954	1.3708E-08	1.7969E-06	2.5273	0.8408
DL-Stachydrine	−6.2069	1.3005E-08	1.7969E-06	2.5306	0.8236
O-Phosphocolamine	−6.4011	5.3319E-09	1.0198E-06	2.5867	0.82
Dodecanedioic acid	−6.3816	5.8349E-09	1.0198E-06	2.5812	0.8196
Cinnamyl alcohol	−6.1635	1.5849E-08	1.9177E-06	2.5179	0.8152
SM 9:1;2O/26:7	−2.6342	0.0098039	0.042658	1.225	0.804
PC O-18:3	−6.2764	9.465E-09	1.4888E-06	2.5509	0.8036
Piperine	−4.6855	9.0083E-06	0.00022141	2.0381	0.8008

### Shared metabolites related to sepsis after trauma under the impact of severe trauma and infection

In the two comparisons (TDS vs. TDDS and sepsis vs. TDS), 26 significantly different metabolites were found to be shared (Table 5). These metabolites may be closely related to the pathogenesis of sepsis. Of which, 19 metabolites belong to lipid metabolism. To be specific, LPC O-22:1, LPC O-22:0, LPC 22:0-SN1, LPC O-24:2, LPC 40:5, and LPC 18:0 are members of lysophosphatidylcholine (LPC) family, PC 17:2_16:4, PC 20:5_22:6, PC O-38:1, PC 38:7, PC 16:1_16:2, PC O-36:2, and PC 16:0_26:6 belong to phosphatidylcholine (PC), PE O-18:2_18:2 is a type of phosphatidylethanolamine (PE), SM 31:1;2O, SM 8:1;2O/22:0, and SM 32:2;2O fall into the sphingomyelin (SM) category.

**Table 5:** 26 Significantly different metabolites shared in the two comparisons (TDS vs. TDDS and sepsis vs. TDS).

Metabolite name	Category
LPC O-22:1	LPCs
LPC O-22:0	
LPC 22:0-SN1	
LPC O-24:2	
LPC 40:5	
PC 17:2_16:4	PCs
PC 20:5_22:6	
PC O-38:1	
PC 38:7	
PC 16:1_16:2	
PC O-36:2	
PC 16:0_26:6	
PE O-18:1_18:2	Phosphatidylethanolamine
SM 31:1;2O	SMs
SM 8:1;2O/22:0	
SM 32:2;2O	
2-Linoleoyl glycerol	Glyceride metabolism
HPK	Unclear
Estriol 17-sulfate	Steroid hormone metabolism
Thiazolidine-4-carboxylic acid	Amino acid metabolism
D-α-Tocopherol	Vitamin metabolism
CAR 20:0	Fatty acid metabolism
2-(tert-butyl)-1,3-thiazolane-4-carboxylic acid	Unclear
(+/−)11(12)-EET	Arachidonic acid metabolism

### Metabolic pathways associated with sepsis after trauma

Patients with post-traumatic sepsis are successively hit by the double blows of trauma and infection. In particular, the metabolic disorders and SIRS caused by severe trauma may lay the groundwork for the occurrence and development of subsequent sepsis. Therefore, we conducted pathway analysis to explore the metabolic pathways related to the occurrence of sepsis under the impact of severe trauma using the differential metabolites obtained from the comparison between the TDS and TDDS groups, and to explore the metabolic pathways related to the occurrence of sepsis under the impact of infection through the differential metabolites from the comparison between the Sepsis and TDS groups.

**Fig. 5:**
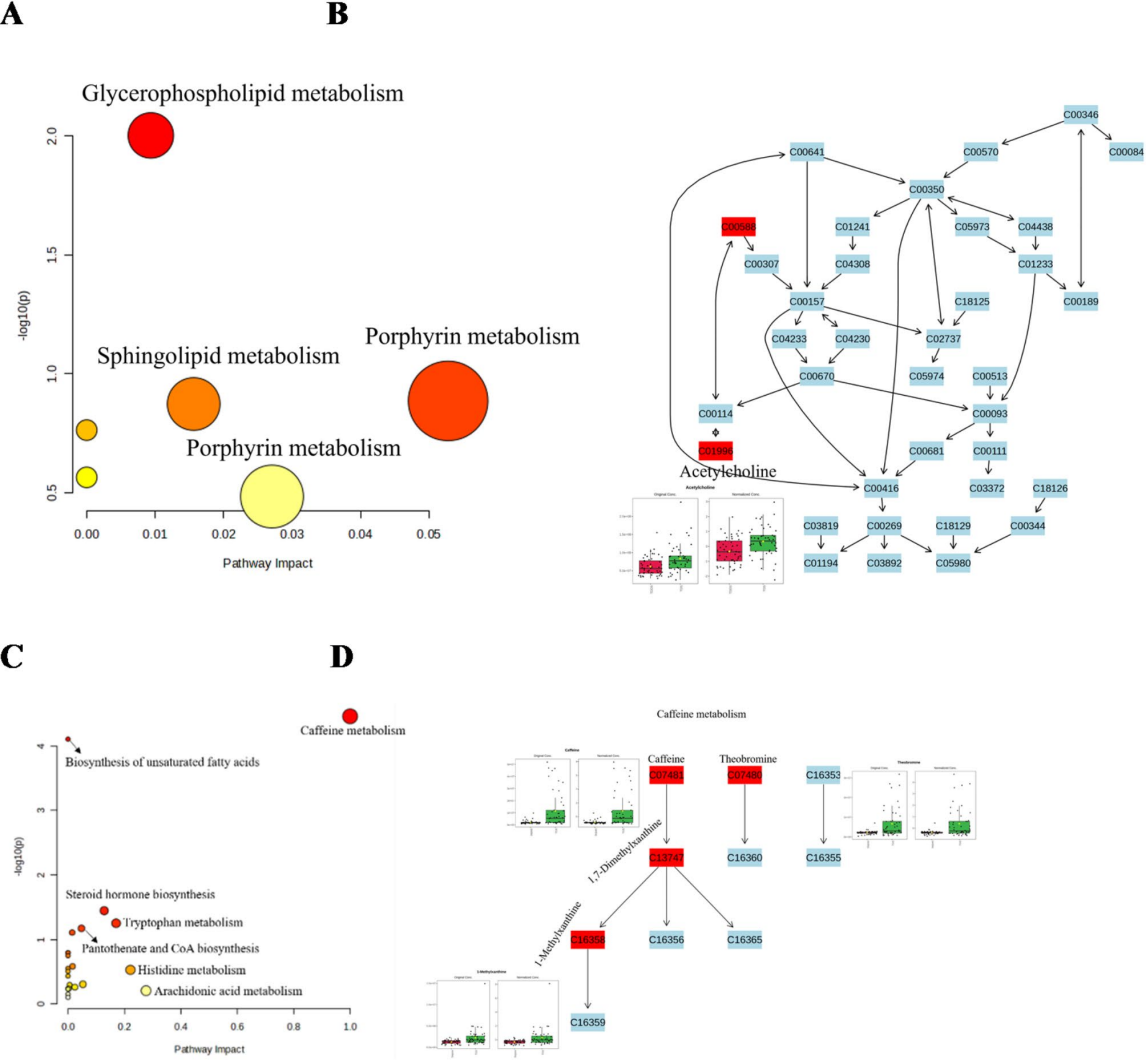
Metabolic pathways associated with sepsis after trauma. (**A**) Metabolic pathways related to sepsis after trauma under the impact of severe trauma. (**B**) Glycerophospholipid metabolism. (**C**) Metabolic pathways associated with sepsis after trauma under the impact of infection (**D**) Caffeine metabolic pathway. *Color gradient and circle size indicate the significance of the pathway ranked by p‑value (yellow: higher p‑value, red: lower p‑value) and pathway impact score (larger circle indicates higher impact score).

Metabolic pathways related to sepsis after trauma under the impact of severe trauma were analyzed through the differential metabolites obtained from the comparison between the TDS and TDDS groups. The top three pathways were: (1) glycerophospholipid metabolism, (2) porphyrin metabolism, and (3) sphingolipid metabolism (Fig. 5A). Among these, glycerophospholipid metabolism was the most significant, with acetylcholine being distinctly higher in theTDS group compared to the TDDS groups (Fig. 5B).Metabolic pathways associated with sepsis after trauma under the impact of infection were analyzed through the differential metabolites obtained from the comparison between the Sepsis group and TDS group, the top three pathways were: (1) caffeine metabolism, (2) biosynthesis of unsaturated fatty acids, and (3) steroid hormone biosynthesis (Fig. 5C). Among these, caffeine metabolism was the most significant, with theobromine, caffeine, and 1-methylxanthine being distinctly lower in the Sepsis group compared to the TDS and Control groups (Fig. 5D).

## Discussion

While trauma is recognized as a risk factor for sepsis, the underlying pathophysiological mechanisms remain poorly understood, and identifying early predictive and diagnostic biomarkers continues to be a challenge. Trauma-induced sepsis is a more complex subtype compared to non-trauma sepsis due to the dual impact of trauma and infection. Additionally, trauma patients may develop SIRS and organ dysfunction, further complicating the early diagnosis of sepsis. In this study, we conducted a prospective observational cohort study to identify early predictive and diagnostic biomarkers for sepsis in trauma patients admitted to the emergency department, as well as to explore metabolic pathways related to trauma-induced sepsis. Most importantly, we collect venous blood from trauma patients within 24 h after injury and conduct follow-ups. Once the patients develop sepsis, we will collect blood samples again. By the comparison between the TDS group and the TDDS group, early predictive biomarkers were screened out to identify the high-risk population prone to developing post-traumatic sepsis. In addition, By the comparison between the Sepsis group and the TDS group, diagnostic biomarkers were screen outed. These diagnostic biomarkers can be used to follow up on the high-risk population, assisting doctors in diagnosing sepsis. In the TDS group, the onset time of sepsis varies from person to person. The timeline for sepsis development after trauma was 1 ~ 14 day [5 (4, 6) day], 46% of patients developed sepsis within 5 days after injury. It is essential to conduct further research that divide the trauma patients into two groups according to the time of sepsis occurrence: the early-onset sepsis group (occurring within 5 days after injury) and the late-onset sepsis group (occurring more than 5 days after injury), to explore the factors and metabolic profiles related to the early occurrence of sepsis.

We identified five key metabolites as early predictive biomarkers for sepsis following trauma from differential metabolites in the TDS vs. TDDS groups: (1) docosatrienoic acid, (2) 7-alpha-carboxy-17-alpha-carboxyethylandrostan lactone phenyl ester, (3) SM 8:1;2O/26:1, (4) N1-[1-(3-isopropenylphenyl)-1-methylethyl]-3-oxobutanamide, and (5) SM 34:2;2O. The ROC curve demonstrated robust predictive capacity (all AUC ≥ 0.94). Docosatrienoic acid, 7-alpha-carboxy-17-alpha-carboxyethylandrostan lactone phenyl ester, and N1-[1-(3-isopropenyl phenyl)-1-methylethyl]-3-oxobutanamide were significantly elevated in both the TDS and Sepsis groups, while SM 8:1;2O/26:1 and SM 34:2;2O were lower compared to the TDDS group (Fig. 2B). Among these differential metabolites, docosatrienoic acid is one of the very long-chain polyunsaturated fatty acids (VLCPUFAs)^38^, SM 8:1;2O/26:1 and SM 34:2;2O belong to sphingomyelins (SMs). The classification of 7-alpha-carboxy-17-alpha-carboxyethylandrostan lactone phenyl ester and N1-[1-(3-isopropenyl phenyl)-1-methylethyl]-3-oxobutanamide remains unconfirmed. Further replication studies are necessary to validate these findings in independent cohorts.Five significant metabolites were identified as early diagnostic biomarkers for sepsis after trauma from the comparison between the Sepsis and TDS groups, included (1) LPC O-22:1, (2) LPC O-22:0, (3) Uric acid, (4) LPC O-24:2, and (5) LPC 22:0-SN1. The ROC curve demonstrated their remarkable diagnostic potential (Fig. 4C). Among these biomarkers, LPC O-22:1, LPC O-22:0, LPC O-24:2, and LPC 22:0-SN1 were classified as LPCs, uric acid is the final product of purine metabolism in humans and higher primates^39^. This study was the first to reveal that decreased uric acid levels increase the risk of sepsis after trauma. The results of some metabolomics of sepsis and sepsis after trauma from other study were shown in Table 6.

**Table 6:** Representation of the results of some metabolomics of sepsis and sepsis after trauma.

Authors	Cases	Controls	Sample type	Metabolites identified
Benjamin J. Blaise et al.^36^. (2013)	*N* = 10 no later sepsis development in traumatized patients	*N* = 12 later sepsis development in traumatized patients	Plasma	Valine, citrate, aspartate, allantoin and hydroxybutyrate were associated with the later development of sepsis.
Ke Feng et al.^37^. (2022)	*N* = 30 multiple trauma complicated with sepsis	*N* = 30 multiple trauma patients	Plasma	The major metabolism pathways related to sepsis after trauma were glycerophospholipid, sphingolipid, tryptophan, pyrimidine, phenylalanine, alanine, aspartate, glutamate, butanoate, pyrimidine, arginine, proline, histidine, and alpha-linolenic acid metabolism pathways.
Youjin Chang et al.^26^. (2023)	*N* = 54 Sepsis‑induced ARDS	*N* = 30 healthy controls	plasma	The main distinguishing metabolites distinct from sepsis‑induced ARDS patients from healthy controls were lysophosphatidylethanolamine plasmalogen, PE plasmalogens, and phosphatidylcholines (PCs).
Qi Chen et al. ^40^. (2022)	*N* = 63 patients with sepsis	*N* = 43 healthy controls	Serum	Dysregulation of amino acid metabolism was related to sepsis. Seventy-three differentially expressed metabolites could predict sepsis.
Han She et al. ^41^ (2023)	*N* = 30 septic patients	*N* = 15 healthy controls	Serum	Significantly altered metabolites were mainly enriched in lipid metabolism-related signaling pathways

Based on pathway analysis, metabolic pathways related to the occurrence of sepsis under the impact of severe trauma and under the impact of infection were identify, the former included glycerophospholipid metabolism, porphyrin metabolism, and sphingolipid metabolism, the later included caffeine metabolism, biosynthesis of unsaturated fatty acids, and steroid hormone biosynthesis. Glycerophospholipids are classified into different groups due to variable substitutions. The crucial ones are PCs, PEs, phosphatidic acids, and phosphatidylinositols^41^. These can act as substrate sources for generating various lyso-phospholipids, like LPCs, lyso-phosphatidylethanolamines, and lyso-phosphatidic acids. It is very interesting that 26 significantly different metabolites were found to be shared in the two comparisons (TDS vs. TDDS and sepsis vs. TDS), these metabolites may be closely related to the pathogenesis of sepsis after trauma, especially, LPCs, PCs, and SMs. Therefore, it is evident that glycerophospholipid metabolism is closely associated with the occurrence and development of post-traumatic sepsis.

VLCPUFAs play a pivotal role in maintaining cell membrane integrity and serve as precursors to bioactive compounds that regulate essential physiological processes^38^. Docosatrienoic acid, an ω-3 VLCPUFA, has demonstrated potent anti-inflammatory and anti-tumor properties^42^. During inflammation and infection, oxylipins-derived from polyunsaturated fatty acids (PUFAs) mediate immune responses by bridging lipid metabolism and immunity, influencing the production of key inflammatory molecules such as prostaglandins, thromboxanes, and lipotoxins^43,44^. These compounds are crucial for clinical research, especially in the regulation of inflammation, thrombosis, and endothelial function, where each system is either stimulated or inhibited by various types of oxylipins^45^. Mitochondrial β-oxidation also acts as a defense mechanism during pathogen invasion. Mariya Misheva et al.^44^. discovered that the suppression of oxylipin levels via β-oxidation influence the regulation of leukocyte function. Docosatrienoic acid exhibits potent anti-inflammatory effects on human macrophages derived from THP-1 monocytes by downregulating the protein expression of pro-inflammatory cytokines^46^. High levels of docosatrienoic acid, as observed in the TDS and Sepsis groups (Fig. 2b), may lead to immune dysregulation by disrupting the balance between pro-inflammatory and anti-inflammatory pathways.

Sphingomyelin, a type of sphingolipid found in cell membranes, particularly abundant in nerve cell membranes and myelin sheaths, plays important roles in maintaining membrane structure, regulating membrane fluidity, and participating in cell signaling processes. Ilias Thomas, et al.^47^ discovered that SMs are closely related to the severity of TBI, in patients with mild TBI, the greatest increase of SMs levels was observed, followed by a decrease as the severity increased. Similarly, SM 8:1;2O/26:1 and SM 34:2;2O were higher in the TDS group with higher ISS compared to the TDDS group. However, Haroon Arshad, et al.^48^ reported that the phospholipid concentrations in patients with community-acquired pneumonia were significantly reduced and returned to normal with clinical improvement, while LPCs, SMs, and ceramides were upregulated. Similar to our study, Jifang Liang et al.^49^. reported SMs in the sepsis group were lower than healthy participants. Amani Al-Mekhlafi et al.^50^. found that the concentration of SM.C16.0 in viral infections was significantly higher compared to non-inflammatory control group, and the concentrations of two SMs (AUC = 0.89, 0.91) were also significantly higher in viral infections than in autoimmune neuroinflammation groups. Their research may show that SMs are good biomarkers in distinguishing between viral infection and aseptic inflammation. Differentiating post-traumatic sterile inflammation from sepsis remains a major challenge, and SMs may be a good option, but further research is needed for verification.

Plasma LPCs are bioactive lipid metabolites of phosphatidylcholine and widely considered as effective pro-inflammatory and harmful mediators, mainly produced by the action of secreted phospholipase A2 (sPLA2) after fatty acids are removed^51,52^. LPCs and lysophosphatidic acid (LPA) are lysophospholipid species regulating acute and chronic inflammatory processes^53^. sPLA2-IIA is particularly elevated in inflammatory processes, triggering the production of bioactive inflammatory mediators and resolution of inflammation^51^. Several studies have shown that LPC are associated with neutrophil priming, immune cell recruitment, and neutrophil extracellular trap (NETs) formation^54–56^. Christopher C. Silliman et al.^54^. found LPCs primed the NADPH oxidase and stimulated multiple neutrophil functions through changing cytosolic calcium. Upasana Parthasarathy et al.^55^. found LPCs were associated with differential expression of mature and immature neutrophil subpopulations in sepsis patients. Hitomi Ohinata, et al.^56^ showed that oxidized phosphatidylcholines and LPC promoted Neutrophil extracellular trap (NETs) formation. It was reported that the upregulation of LPC may lead to allergic skin inflammation by increasing IL17 expression and recruiting neutrophils through the G2A receptor^57^. LPCs are also good biomarkers for distinguishing between bacteremia and non-bacteremia patients, 12 LPCs significantly differed between patients with bacteremia and those without, and the total LPCs level was elevated in patients with bacteremia^58^. Interestingly, similar to WBC and ANC (Table 2), the concentrations of LPCs in the Sepsis group were lower than in the TDS group (Fig. 4A), which may be associated with a state of immunological fatigue following trauma. Some LPCs were associated with anti-inflammatory. Daniel Hornburg et al.^59^. observed an increased abundance of LPCs during inflammation, hence patients were healthier. A study suggested that LPC mitigates lung inflammation induced by sepsis through adjusting neutrophil function, reducing the infiltration of innate immune cells into the lungs, decreasing the migration of neutrophils in inflammatory conditions, and enhancing the phagocytosis of damaged lungs^60^. In summary, LPCs exhibit both pro-inflammatory and anti-inflammatory functions and are implicated in the development of sepsis following trauma. However, the precise mechanisms underlying their role in this process require further investigation.

Uric acid, a well-known DAMP molecule, exhibits dual roles: acting as an antioxidant by scavenging reactive oxygen species (ROS) and as a pro-oxidant by generating ROS^61,62^. Its influence on inflammation varies among studies. Masafumi Kurajoh et al. demonstrated that in individuals without hyperuricemia, low uric acid levels may exacerbate COVID-19 progression by heightening inflammation^61^. Conversely, Qiuyue Ma et al.^63^. found that soluble uric acid impairs neutrophil migration and phagocytosis by disrupting β2 integrin activity. Soluble uric acid also enhances autophagic flux in infected macrophages, increasing IL-1β production during bacterial infection^64^. Xingyong Wan et al.^65^. reported that uric acid activates the NLRP3 inflammasome, while its reduction via allopurinol inhibits this activation. Blocking uric acid synthesis has been shown to modulate infection severity. Wendy Fonseca et al.^62^. revealed that uric acid serves as a key immunomodulatory molecule during respiratory syncytial virus (RSV) infection, inhibition of the uric acid pathway with xanthine oxidase inhibitors (XOI) reduced IL-33 expression, and in RSV-infected mice, XOI treatment decreased lung mucus, innate lymphoid cell 2 counts, macrophages, and IL-33 levels in bronchoalveolar lavage fluid. However, other studies have indicated that the combined use of allopurinol in experimental sepsis worsens outcomes, leading to death, kidney damage, elevated ROS, and increased pro-inflammatory cytokines^66^. Additionally, Yujun Qin et al. identified a causal relationship between uric acid levels and sepsis. Overall, uric acid has both pro-inflammatory and anti-inflammatory properties, though the reasons for this variability remain unclear. Uric acid’s role in sepsis following trauma has received limited clinical attention, and it is rarely included in early predictive studies. Thus, further research is warranted to explore uric acid’s potential as an early diagnostic biomarker for sepsis after trauma.

In our study, glycerophospholipid metabolism was significant metabolic pathway related to sepsis after trauma under the impact of severe trauma and the impact of infection. Shanping Wang et al.^67^. reported isosteviol sodium reduced multiple organ injury in sepsis mice by regulating of glycerophospholipid metabolism and reducing macrophage-driven inflammation. Ziwen Yuan et al.^68^. found that huang-lian-Jie-du decoction can significantly inhibit glycerophospholipid metabolic pathway to reduce the disease activity index of ulcerative colitis mice through inhibiting COX-2 protein expression and PLA2, 5-LOX activity. The study by Fanmei Zou et al.^69^. found that XueBiJing improves sepsis-induced acute lung injury by reducing glycerophospholipid metabolism, phospholipid metabolism, and lipid metabolism associated with ferroptosis. It can be seen that glycerophospholipid metabolism is involved in the occurrence of various diseases by regulating inflammation, immunity and cell death. Our study found that, compared with the TDDS group, the glycerophospholipid metabolism was highly expressed in the TDS group, which might lead to an excessive inflammatory response in the early stage of trauma. Interestingly, the levels of LPCs were low after the onset of sepsis. It may suggest that the excessive inflammatory response in the early stage could result in a subsequent immunosuppressive state, making patients susceptible to sepsis.

PCs, a crucial structural element of the cell membrane, is essential for lipid-dependent signaling pathways^70^. PCs is one of lipid molecular profiles, with anti-inflammatory and pro- inflammatory functions^71,72^. Campos AM, et al.^72^. found that higher expression of PC(36:1) and PC(38:4) along with a decrease in PC(40:6) level were observed under pro-inflammatory environment. Xue Zhang, et al. verified that PC (18:1/18:1) mediated lipopolysaccharide-induced inflammation in HepG2 cell^73^. Due to the impacts of tissue injury, ischemia-hypoxia, and ischemia-reperfusion, sterile SIRS often occurs in the early stage of trauma. Although PCs are important inflammatory regulators, different PCs have different functions. Our research has only screened out some metabolites related to the occurrence of traumatic sepsis, and further animal experiments are required to explore the specific mechanisms.In this study, caffeine metabolism emerged as the most significant pathway related to sepsis after trauma under the impact of infection. Primary metabolites of caffeine metabolism include theobromine, paraxanthine, caffeine, 7-methylxanthine, 1,7-dimethyluric acid, and 1-methylxanthine^74^. Caffeine metabolism is influenced by numerous factors such as genetic determinants, age, sex, pregnancy, medications, and diseases^75^. Caffeine has been shown to alleviate various inflammatory conditions by modulating major cellular and molecular immune components^76^. Persistent downregulation of caffeine metabolism post-trauma may contribute to immunosuppression, increasing susceptibility to sepsis. While research has linked caffeine metabolism to tachyarrhythmias, Alzheimer’s disease, gout, and myocardial infarction, its connection to sepsis after trauma remains understudied^77,78^. A study highlighted caffeine metabolism as a notable pathway in patients with biliary obstruction infected by Clonorchis sinensis compared to non-infected individuals^78^. Additionally, Wei Li et al.^79^. found that hypertensive intracerebral hemorrhage is associated with increased inflammation and downregulation of metabolic pathways, particularly those involving 3,7-dimethyluric acid and 7-methylxanthine in caffeine metabolism. Qianling Jiang et al.^80^. reported that caffeine metabolism is significantly altered following *Klebsiella pneumoniae* infection. Caffeine metabolism was also identified as a distinct metabolic pathway in sepsis patients compared to healthy volunteers^81^. Collectively, these findings suggest that caffeine metabolism plays a critical role in the onset of infection and sepsis and may offer new insights into mechanisms, prevention, and treatment for sepsis after trauma.

This study had several limitations. First, the lack of dynamic sampling prevented the assessment of metabolite changes over time, limiting the understanding of their progression in sepsis. Second, while potential biomarkers for early prediction and diagnosis of sepsis were identified, the absence of inflammation markers meant that their correlation with these biomarkers could not be validated. Third, trauma causes and damage to different organs after injury may impact metabolic profiles. Fourth, we can only collect blood samples. The changes in blood metabolites cannot reflect the metabolic changes within organs. Therefore, the selected metabolites are mainly used as predictive and diagnostic biomarkers. Subsequent animal experiments are required to study the metabolic changes in organs and further explore the pathophysiological mechanisms underlying the development of sepsis after trauma. Finally, given the challenges in diagnosing sepsis, even though blood samples were collected on the first day of diagnosis, there may be discrepancies between the actual onset of sepsis and the time of diagnosis.

## Conclusion

Despite these limitations, this study identified docosatrienoic acid, 7-alpha-carboxy-17-alpha-carboxyethylandrostan lactone phenyl ester, SM 8:1;2O/26:1, N1-[1-(3-isopropenylphenyl)-1-methylethyl]-3-oxobutanamide, and SM 34:2;2O as potential biomarkers for early prediction. Additionally, LPC O-22:1, LPC O-22:0, uric acid, and LPC O-24:2 showed promise in the early diagnosis of sepsis after trauma. Metabolites shared between two comparisons (TDS vs. TDDS and sepsis vs. TDS) may be closely related to the pathogenesis of sepsis, such as LPCs, phosphatidylcholines (PCs), phosphatidylethanolamine (PE), and SMs. Besides, we found glycerophospholipid metabolism and caffeine metabolism were significant pathway related to sepsis after trauma. This study provides a basis for further research into the development of onset and development, early prevention, diagnosis, and treatment of sepsis after trauma based on these metabolites and metabolic pathways.

## Data Availability

Data are available upon request from Ke Feng (Email: fengkedoct@163.com).

## Abbreviations

SIRS: Systemic inflammatory response syndrome
UHPLC-MS/MS: Ultrahigh-performance liquid chromatography-tandem mass spectrometer
AUC: Area under the curve
CI: Confidence interval
SM: Sphingomyelin
LPC: Lysophosphatidylcholine
DAMPs: Damage-associated molecular patterns
ISS: Injury severity score
GCS: Glasgow coma scale
T: Temperature
HR: Heart rate
INR: International normalized ratio
CRP: C-reaction protein
ROC: Receiver operating characteristic
PCT: Procalcitonin
OR: Odd ratio
AIS: Abbreviated injury scale
MV: Mechanical ventilation
DVC: Deep vein catheterization
SOFA: Sequential organ failure assessment
WBC: White blood cell count
RR: Respiratory rate
SBP: Systolic blood pressure
DBP: Diastolic blood pressure
SPO_2_: Oxyhemoglobin saturation
ICU: Intensive care unit
LOS: Length of stay
PLS-DA: Partial least squares discriminant analysis
VIP: Variable importance of the projection
FDR: False discovery rate
VLCPUFA: Very long-chain polyunsaturated fatty acid
PUFAs: Polyunsaturated fatty acids
ROS: Reactive oxygen species
RSV: Respiratory syncytial virus
